# Dietary Selenium Supplementation Modulates Growth of Brain Metastatic Tumors and Changes the Expression of Adhesion Molecules in Brain Microvessels

**DOI:** 10.1007/s12011-015-0595-x

**Published:** 2015-12-26

**Authors:** Jagoda K. Wrobel, Gretchen Wolff, Rijin Xiao, Ronan F. Power, Michal Toborek

**Affiliations:** Department of Biochemistry and Molecular Biology, University of Miami Miller School of Medicine, Miami, FL 33136 USA; Nutrigenomics Research Center, Alltech, Nicholasville, KY 40356 USA; Jerzy Kukuczka Academy of Physical Education, Katowice, 40-065 Poland

**Keywords:** Selenium, Brain, Tumor progression, Adhesion, Cell adhesion molecules, NF-κB

## Abstract

Various dietary agents can modulate tumor invasiveness. The current study explored whether selenoglycoproteins (SeGPs) extracted from selenium-enriched yeast affect tumor cell homing and growth in the brain. Mice were fed diets enriched with specific SeGPs (SeGP40 or SeGP65, 1 mg/kg Se each), glycoproteins (GP40 or GP65, 0.2–0.3 mg/kg Se each) or a control diet (0.2–0.3 mg/kg Se) for 12 weeks. Then, murine Lewis lung carcinoma cells were infused into the brain circulation. Analyses were performed at early (48 h) and late stages (3 weeks) post tumor cell infusion. Imaging of tumor progression in the brain revealed that mice fed SeGP65-enriched diet displayed diminished metastatic tumor growth, fewer extravasating tumor cells and smaller metastatic lesions. While administration of tumor cells resulted in a significant upregulation of adhesion molecules in the early stage of tumor progression, overexpression of VCAM-1 (vascular call adhesion molecule-1) and ALCAM (activated leukocyte cell adhesion molecule) messenger RNA (mRNA) was diminished in SeGP65 supplemented mice. Additionally, mice fed SeGP65 showed decreased expression of acetylated NF-κB p65, 48 h post tumor cell infusion. The results indicate that tumor progression in the brain can be modulated by specific SeGPs. Selenium-containing compounds were more effective than their glycoprotein controls, implicating selenium as a potential negative regulator of metastatic process.

## Introduction

Brain metastatic tumors are detected in 10–30 % of all cancer patients. However, the incidence rates of brain metastasis are increasing due to improved diagnostic methods and better control of primary tumors, resulting in longer patient survival [1, 2]. The treatment of brain metastasis requires a multidisciplinary team approach, but therapeutic options for patients affected by the disease are still limited. Although our understanding of the process of brain metastasis formation remains incomplete, the interactions between tumor cells and the brain microenvironment are considered to be critical determinants in their progression towards metastasis, dormancy, or clearance [3, 4]. Principal in these events is the expression of cell adhesion molecules (CAMs) on the surface of the brain endothelium. Cell adhesion molecules are cell surface glycoproteins that are typically transmembrane receptors mediating cell-cell or cell-matrix interactions and influencing tumor cell adhesion, migration, and metastatic potential [3–5].

Selenium (Se) is an essential micronutrient with complex effects on human biology. Although widely distributed throughout the body, Se is particularly well maintained in the brain; even upon severe dietary Se deficiency, Se levels remain high in the brain as compared to other tissues [6, 7]. Importantly, changes in Se concentration in the blood and the brain have been reported in patients suffering from brain diseases, including brain tumors [8, 9].

Experimental in vivo and in vitro studies have demonstrated that supplemental Se could reduce the risk of certain types of cancer and modulate many processes relevant to tumor biology [9–11]. Although it is likely that Se acts through multiple pathways, the exact molecular mechanisms by which antitumor activity of Se compounds are mediated remain unclear [11, 12]. The functions of Se in human physiology are largely mediated by selenoproteins (25 of such proteins are identified so far), in which Se is incorporated as selenocysteine [13, 14]. Although the functions of most of these proteins are still being investigated, approximately half of them have been shown to participate in oxidoreductase reactions [14]. Indeed, it has been demonstrated that Se incorporated into selenoproteins reduces oxidative stress and limits DNA damage, both of which are associated with decreased cancer risk [11, 15]. Other possible mechanisms that may be involved in the anticancer and antimetastatic properties of Se include alteration of carcinogen metabolism, modulation of inflammatory and immune responses, regulation of the cell cycle, induction of apoptosis of cancer cells, alterations of DNA methylation, and inhibition of angiogenesis and tumor cell motility [11, 12, 15–18]. The efficacy of Se supplementation is dependent on the dose and chemical form of Se, as distinct forms of Se in various concentrations can express dramatically different biological effects [11, 18, 19]. Moreover, research suggests that only patients with a low Se status may benefit from additional Se intake, while individuals whose plasma Se concentration is 122 μg/L or higher could be adversely affected and should not be supplemented with Se [19, 20].

Se-enriched yeast is the most common source of Se available commercially and is also the most widely used Se-supplementation method in clinical trials [21]. We have reported previously that dietary supplementation with selenized yeasts markedly decreased growth of brain metastatic tumors in mice [18]. We demonstrated in a subsequent in vitro study that specific selenoglycoproteins extracted from Se-enriched yeasts have the ability to attenuate adhesion of tumor cells to human brain endothelium [22]. The aim of the current study was to evaluate if selected selenoglycoprotein fractions could modulate growth of brain metastatic tumors in vivo. We show that tumor progression in the brain can be modulated by supplementation with specific selenoglycoproteins. Decreased activity of NF-κB and reduced expression of cell adhesion molecules may be one of the mechanisms responsible for the observed effects.

## Materials and Methods

### D122 Lewis Lung Carcinoma Cells

D122 Lewis lung carcinoma cells tagged with luciferase (D122-Luc) were maintained in Dulbecco’s modified Eagle’s medium (DMEM) GlutaMax medium (Life Technologies, Carlsbad, CA, USA), supplemented with 10 % fetal bovine serum (Thermo Scientific, Rockford, IL, USA) and 1 % penicillin/streptomycin (Life Technologies). The cells were transfected with green fluorescent protein (GFP)-encoding vector (pBMN-GFP; Orbigen, San Diego, CA, USA) to produce D122-Luc/GFP cells. D122-Luc/GFP cells were selected by treatment with 3 μg/mL puromycin, followed by sorting of fluorescent cells. Cells were maintained in 1 μg/mL puromycin-contained media. Cells were used for immunostaining until the fifth passage.

### Animal Dietary Protocol

Selenoglycoproteins were extracted from selenium-enriched yeast at pH 4.0 and 6.5 (the fractions are called SeGP40 and SeGP65, respectively). Control treatments included similarly extracted glycoproteins from normal yeast, called GP40 and GP65. Male C57BL/6 mice, 12 weeks of age (Jackson Labs, Bar Harbor, ME, USA) were fed diets enriched with SeGP40, SeGP65 (each containing 1 mg Se/kg), GP40, GP65 (each containing 0.2–0.3 mg Se/kg), or a control diet (0.2–0.3 mg Se/kg) for 12 weeks. Se supplementation, especially in the form of organic forms, results in significant enrichment in the brain [23]. Experimental diets were prepared based on Teklad 2016 Base Diet by Harlan Laboratories (Indianapolis, IN, USA), followed by an analysis of the final selenium content.

All diets were provided ad libitum. SeGP and GP fractions were obtained as described previously [22]. Briefly, the process of SeGPs extraction from standardized Se-enriched yeast was designed in order to capture and concentrate as many water-soluble SeGPs as possible based upon the main pH ranges of these species, namely pH 4.0 (SeGP40) and pH 6.5 (SeGP65). The organic Se content, in the Se-enriched yeast used for the extraction of the SeGPs was equal to or greater than 98 % of total Se content. GP fractions were obtained in the same manner from non-Se-enriched yeast. Diets without Se enrichment (i.e., GP40 and GP65) were used as controls for SeGP40 and SeGP65, respectively, in order to evaluate the effect of Se and/or glycoprotein components. All tested diets were isocaloric (3.1 Kcal/g) and equal in terms of the content of protein, fat, and carbohydrates (16.2, 3.6, and 52.8 %, respectively). Diet composition is provided in Table 1.

**Table 1:** Experimental diets

	Control	SeGP40	GP40	SeGP65	GP65
Macronutrients					
Protein (% by weight)	16.2	16.2	16.2	16.2	16.2
Carbohydrate (% by weight)	52.8	52.8	52.8	52.8	52.8
Fat (% by weight)	3.6	3.6	3.6	3.6	3.6
Calorie content (Kcal/g)	3.1	3.1	3.1	3.1	3.1
Fiber (%)	3.3	3.3	3.3	3.3	3.3
Minerals					
Calcium (%)	1.0	1.0	1.0	1.0	1.0
Non-phytate phosphorus (%)	0.4	0.4	0.4	0.4	0.4
Sodium (%)	0.2	0.2	0.2	0.2	0.2
Potassium (%)	0.6	0.6	0.6	0.6	0.6
Chloride (%)	0.4	0.4	0.4	0.4	0.4
Magnesium (%)	0.2	0.2	0.2	0.2	0.2
Zinc (mg/kg)	70	70	70	70	70
Manganese (mg/kg)	100	100	100	100	100
Copper (mg/kg)	15	15	15	15	15
Iodine (mg/kg)	6	6	6	6	6
Iron (mg/kg)	200	200	200	200	200
Selenium (mg/kg)	0.2–0.3	1.2–1.3	0.2–0.3	1.2–1.3	0.2–0.3
Amino acids					
Aspartic acid (%)	1.0	1.0	1.0	1.0	1.0
Glutamic acid (%)	3.3	3.3	3.3	3.3	3.3
Alanine (%)	0.9	0.9	0.9	0.9	0.9
Glycine (%)	0.7	0.7	0.7	0.7	0.7
Threonine (%)	0.6	0.6	0.6	0.6	0.6
Proline (%)	1.5	1.5	1.5	1.5	1.5
Serine (%)	0.8	0.8	0.8	0.8	0.8
Leucine (%)	1.9	1.9	1.9	1.9	1.9
Isoleucine (%)	0.7	0.7	0.7	0.7	0.7
Valine (%)	0.8	0.8	0.8	0.8	0.8
Phenylalanine (%)	0.9	0.9	0.9	0.9	0.9
Tyrosine (%)	0.5	0.5	0.5	0.5	0.5
Methionine (%)	0.3	0.3	0.3	0.3	0.3
Cystine (%)	0.3	0.3	0.3	0.3	0.3
Lysine (%)	0.8	0.8	0.8	0.8	0.8
Histidine (%)	0.4	0.4	0.4	0.4	0.4
Arginine (%)	0.8	0.8	0.8	0.8	0.8
Tryptophan (%)	0.2	0.2	0.2	0.2	0.2
Vitamins					
Vitamin A (IU/g)	15.0	15.0	15.0	15.0	15.0
Vitamin D3 (IU/g)	1.5	1.5	1.5	1.5	1.5
Vitamin E (IU/kg)	110.0	110.0	110.0	110.0	110.0
Vitamin K3 (mg/kg)	50.0	50.0	50.0	50.0	50.0
Vitamin B1 (mg/kg)	17.0	17.0	17.0	17.0	17.0
Vitamin B2 (mg/kg)	15.0	15.0	15.0	15.0	15.0
Niacin (mg/kg)	75.0	75.0	75.0	75.0	75.0
Vitamin B6 (mg/kg)	18.0	18.0	18.0	18.0	18.0
Pantothenic Acid (mg/kg)	33.0	33.0	33.0	33.0	33.0
Vitamin B12 (mg/kg)	0.1	0.1	0.1	0.1	0.1
Biotin (mg/kg)	0.4	0.4	0.4	0.4	0.4
Folate (mg/kg)	4.0	4.0	4.0	4.0	4.0
Choline (mg/kg)	1030	1030	1030	1030	1030
Fatty acids					
C16:0 palmitic (%)	0.5	0.5	0.5	0.5	0.5
C18:0 stearic (%)	0.1	0.1	0.1	0.1	0.1
C18:1ω9 oleic (%)	0.7	0.7	0.7	0.7	0.7
C18:2ω6 linoleic (%)	2.0	2.0	2.0	2.0	2.0
C18:3ω3 linolenic (%)	0.1	0.1	0.1	0.1	0.1
Total saturated (%)	0.6	0.6	0.6	0.6	0.6
Total monosaturated (%)	0.7	0.7	0.7	0.7	0.7
Total polyunsaturated (%)	2.1	2.1	2.1	2.1	2.1
Other					
Cholesterol (mg/kg)	–	–	–	–	–

Body mass and food intake were monitored once a week during the entire feeding period. Mice were euthanized with carbon dioxide followed by decapitation. All procedures, which complied with the guidelines of the American Association for Accreditation of Animal Care (AAALAC), were approved by the University of Miami Institutional Animal Care and Use Committee.

### Tumor Cell Infusion, Timeline, and Bioluminescent Imaging

At the conclusion of the feeding period, mice were randomly assigned to vehicle or tumor groups. Mice were anesthetized with isoflurane and 1.0 × 10^6^ Lewis lung carcinoma D122-Luc/GFP cells were slowly infused into the brain circulation. The control/vehicle group was infused with cell culture medium alone. Using the procedure standardized in our laboratory [24], the common carotid artery was isolated along with the internal and external carotid arteries. A 6-0 silk suture was used to ligate the end of the external carotid artery distal to the bifurcation point while the internal carotid artery and common carotid artery were temporarily closed with vessel clips. A small incision was made in the external carotid artery proximal to the bifurcation point of the common carotid artery and tubing was inserted into the common carotid artery. After the vessel clip was removed from the internal carotid artery, tumor cell suspension or the vehicle was infused. The tubing was removed and the external carotid artery was closed with a second suture and the remaining vessel clip removed. Blood flow returned and the surgical site was cleaned and closed.

The outcomes of the dietary supplementation and tumor cell infusion were evaluated using two time points. For the short-term studies, mice were euthanized 48 h post tumor cell infusion, which coincides with transcapillary extravasation of tumor cells. The second time point was 3 weeks post tumor cell infusion (long-term studies), when brain metastases are fully developed.

In the long-term study, the development of brain metastatic nodules was monitored once a week using the IVIS Xenogen Bioluminescence Imager (Caliper Life Sciences, Hopkinton, MA, USA). Prior the measurements, both tumor and control (vehicle) animals were anesthetized with isoflurane in oxygen and injected ip with D-luciferin potassium salt (2 mg/100 μL) to induce bioluminescence of D122-Luc cells. Identical instrument settings were used for all measurements.

### Plasma Selenium Concentration

Selenium content was analyzed using an inductively coupled plasma mass spectrometer (ICP-MS) Agilent 7700 (Agilent Technologies, Inc., Tokyo, Japan). Briefly, blood was collected by cardiac puncture into heparinized tubes and centrifuged at 4 °C for 15 min at 1000×*g*, followed by an additional spin at 10,000×*g* at 4 °C for 10 min for complete platelet removal. All standards and samples were prepared in buffer solution (4 % butanol, 2 % ammonium hydroxide, 0.1 % EDTA, 0.1 % Triton X-100). Buffer solution was dispensed into 15 mL centrifuge tubes, and 250 μL of plasma was added. Samples were analyzed in duplicate. Three replicates of reference material “Plasma Control” for trace elements, lyophilized, Level 1 (Se certified value of 80 ng/mL ± 16 ng/mL, ClinChek®, RECIPE, Munich, Germany) were prepared using the same procedure, and were recovered at 98.72 %. Signal intensity was compared to an external calibration curve prepared from a 1000 μg/mL Se standard (SCP-Science, New York, NY, USA) over a 0.2–64 ng/mL dynamic range (*R*^2^ = 0.9993). Diluted Rhodium (Rh, SCP-Science) was used as internal standard at a concentration of 1 μg/mL.

### Isolation of Brain Microvessel-Enriched Fraction

Brain microvessels were isolated as described earlier [25] with modifications. The method allows for isolation of microvessel-enriched fraction, with some minimal contamination from other cell types. Following decapitation, brains were extracted and immediately immersed in ice-cold PBS. The choroid plexus, meninges, cerebellum, and brain stem were removed and the hemispheres were separated. The hemisphere ipsilateral to tumor cell infusion was homogenized in 2 mL of ice-cold isolation buffer [102 mM NaCl, 4.7 mM KCl, 2.5 mM CaCl_2_, 1.2 mM KH_2_PO_4_, 1.2 mM MgSO_4_, 15 mM HEPES, 25 mM NaHCO_3_, 10 mM glucose, 1 mM Na pyruvate; pH 7.4 with Complete Protease Inhibitor (Roche, Indianapolis, IN, USA)] and filtered through a 300-μm mesh filter (Spectrum Laboratories, Rancho Dominguez, CA, USA). Then, 8 mL of 26 % dextran (molecular weight 150 kD, Sigma, St. Louis, MO, USA) was added to the homogenates, mixed well and samples were centrifuged at 5800×*g* for 20 min at 4 °C. The supernatants were discarded, and the pellets were resuspended in isolation buffer, filtered through a 120-μm mesh filter (EMD Millipore, Billerica, MA, USA) and repelleted by centrifugation (1500×*g* for 10 min at 4 °C). Isolated microvessels were prepared for immunofluorescent analysis or RNA extraction.

### Immunofluorescent Staining

Brain microvessels were smeared on slides, fixed for 10 min at 98 °C and then for additional 10 min at room temperature in 4 % paraformaldehyde. After washing 3 times with chilled PBS, slides containing isolated microvessels were permeablized with 0.1 % Triton X-100 (Sigma) for 30 min at room temperature, then rinsed with PBS. Slides were blocked using 3 % bovine serum albumin (BSA) in PBS for 1 h at room temperature followed by overnight incubation at 4 °C with anti-NF-κB p65 (acetyl K310, Abcam, Cambridge, MA, USA) at 1:200 dilution. Following washes with PBS, α-rabbit Alexa Fluor 488 (Invitrogen, Camarillo, CA, USA) was applied at 1:400 dilution in 3 % BSA in PBS. After 90 min of incubation at room temperature, protected from light, the slides were washed and stained with DAPI (Cell Signaling, Danvers, MA, USA) at 1 μg/mL concentration in PBS, to visualize the nuclei. Slides were mounted with ProLong® Diamond Antifade Mountant (Life Technologies). Images were acquired using a Nikon Eclipse Ti-U fluorescence microscope and the NES Elements software.

### Real-Time Quantitative Reverse Transcription PCR

RNA was extracted from brain microvessels using TRIzol reagent (Life Technologies) according to the manufacturer’s protocol. Reverse transcription was performed using the Reverse Transcription System (Promega, Madison, WI, USA). ICAM-1, VCAM-1 and ALCAM messenger RNA (mRNA) levels were assessed using a 7500 Real Time PCR System (Applied Biosystems, Foster City, CA, USA) and TaqMan Universal PCR Master Mix. The primers and probes were obtained from Applied Biosystems. The following thermocycling conditions were used: 50 °C for 2 min, followed by 95 °C for 10 min, 95 °C for 15 s, and 60 °C for 1 min for 40 cycles. The threshold cycle (*C*_T_) from each well was determined using 7500 Software v2.0.5 from Applied Biosystems. Expression of mRNA was calculated by the comparative *C*T method. PCR amplification of GAPDH (housekeeping gene) was performed for each sample to normalize mRNA levels of the target genes. All samples were prepared in triplicate.

### Immunohistochemistry

Brains were fixed in 10 % formalin (EMD Millipore), embedded into paraffin, and cut into a series of 5-μm thick sections with 150-μm thick intersections. Sections were mounted onto glass slides, deparaffinized, and subjected to antigen retrieval in citrate buffer (pH 6.0). Endogenous peroxidase activity was inhibited by treatment with 3 % H_2_O_2_ in methanol for 20 min. The slides were rinsed in Tris-buffer saline containing 0.1 % Triton X-100 (TBST), blocked with 5 % rabbit serum in TBST, and incubated with chicken anti-GFP monoclonal antibody (1:400; Aves Lab, Tigard, OR, USA) at 4 °C overnight, followed by incubation with rabbit anti-chicken IgY-HRP secondary antibody (1:200; Abcam) for 1.5 h at room temperature. Diaminobenzidine (Vector Laboratories, Burlingame, CA, USA) was used for color developing. The sections were counterstained with hematoxylin (Sigma) and examined under a light microscope (Nikon, Melville, NY, USA).

### Statistical Analysis

The data were analyzed by one-way analysis of variance (ANOVA) or two-way ANOVA, with Fisher’s least significant difference (LSD) test. Data were further evaluated using Dixon’s *Q* test. GraphPad Prism software (La Jolla, CA, USA) was used for the analyses. Statistical probability of *p* < 0.05 was considered significant.

## Results

### Body Mass and Dietary Consumption of Different Selenoglycoprotein/Glycoprotein Fractions

**Fig. 1:**
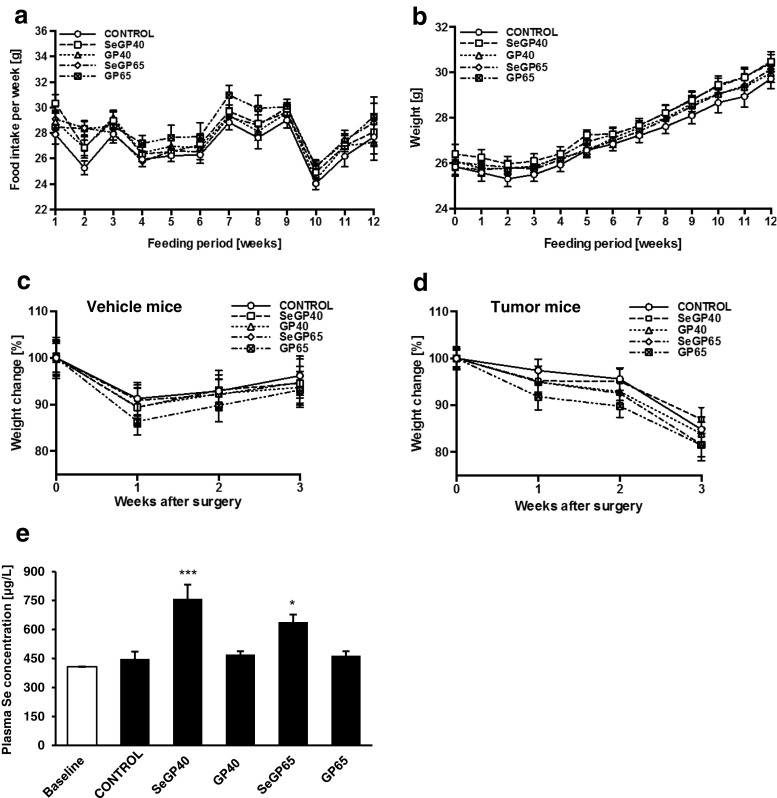
Body mass, dietary consumption of different selenoglycoprotein/glycoprotein fractions and plasma Se concentrations. Mice were fed experimental diets enriched with SeGP40, G40, SeGP65, GP65, or a control diet for 12 weeks. At the conclusion of the feeding period, mice were infused with 1.0 × 10^6^ D122-Luc/GFP cells in culture medium (tumor mice) or with culture medium alone (vehicle mice). **a** Food intake and **b** weight change were monitored once a week during the feeding period. After surgery, body mass was measured once per week up to the third week in **c** vehicle or **d** tumor-infused mice. **e** Plasma Se concentration measured before the dietary supplementation (Baseline) and after 12 week feeding period with indicated diets. Values are mean ± SEM., *n* = 32 mice per group (*a* and *b*), *n* = 10 mice per group (*c* and *d*), *n* = 4 (*e*). Significantly different as compared with control at ***p < 0.001 or *p < 0.05, one-way ANOVA

Mouse food intake and body mass were monitored once a week during the entire 12-wk feeding period (Fig. 1a–b). Although the average food intake varied from week to week, there were no significant differences between the experimental groups (Fig. 1a). The plasma concentration of Se was measured before dietary supplementation and at the end of the study. Baseline Se plasma concentration was ∼400 μg/L; however, a 12-week supplementation with SeGP40 and SeGP65 increased the concentration to 630–880 μg/L and 580–730 μg/L, respectively (Fig. 1e). Mice receiving diets supplemented with selenoglycoproteins had significantly higher plasma Se levels.

Starting from week 4, the animals demonstrated progressive weight gain and at the end of the feeding period all the animals, regardless of diet, weighed approximately 15 % more than at week 0. There were no significant differences in animal weight among the experimental and control groups (Fig. 1b).

During the first week after the surgery both vehicle and tumor mice lost between 3 to 13 % of their body mass (Fig. 1c–d). During the following 2 weeks, vehicle infused mice increased their body mass, reaching to 90–95 % of their initial weight by the third week (Fig. 1c). However, the body mass of tumor cell infused mice continued to decrease, independent of diet, reaching 82–87 % of their initial weight on week 3 (Fig. 1d).

### Selenoglycoprotein Fraction SeGP65 Attenuates Growth of Metastatic Tumors in the Brain

**Fig. 2:**
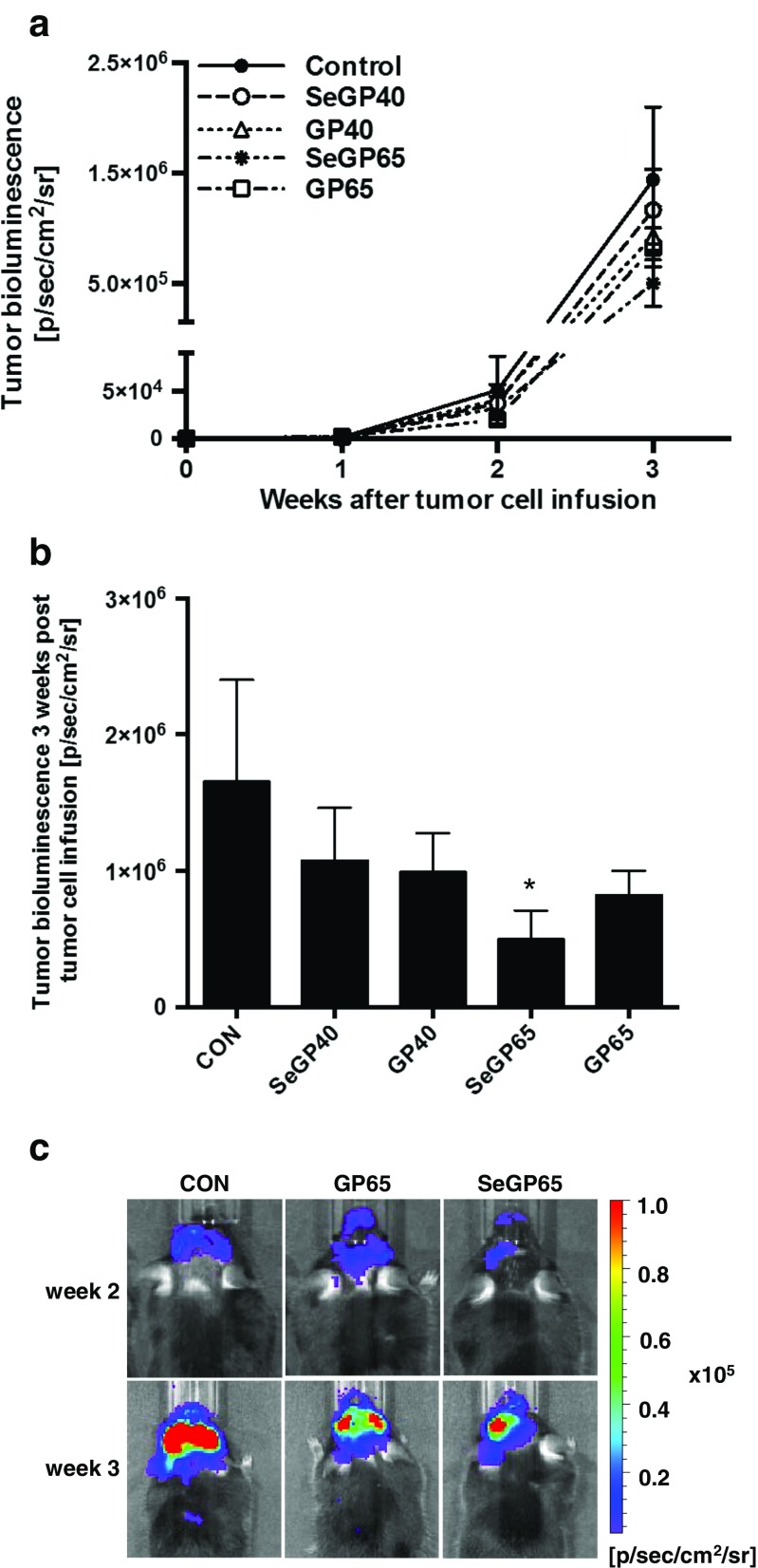
In vivo monitoring of brain metastatic progression. The development of brain metastatic nodules was monitored each week using the IVIS Xenogen Bioluminescence Imager based on the bioluminescent signal from D122-Luc/GFP tumor cells. **a** Quantified results of tumor progression over 3 weeks of observation. **b** Quantified bioluminescent signal for each dietary group at the third (final) week after tumor cell infusion. **c** Representative images at weeks 2 and 3 after tumor cell infusion. Values are mean ± SEM., *n* = 13–15 mice per group. *Significantly different as compared with mice fed control diet at *p < 0.05, one-way ANOVA

In the long-term studies, the development of brain metastatic nodules was monitored each week using live imaging. During the first 2 weeks, brain tumor bioluminescence progressed similarly in all experimental groups (Fig. 2a). At the third week of monitoring, mice exposed to SeGP65 enriched diet showed significantly diminished metastatic tumor growth as compared to control (Fig. 2b-c). Mice that were not infused with tumor cells exhibited bioluminescence at the background levels.

**Fig. 3:**
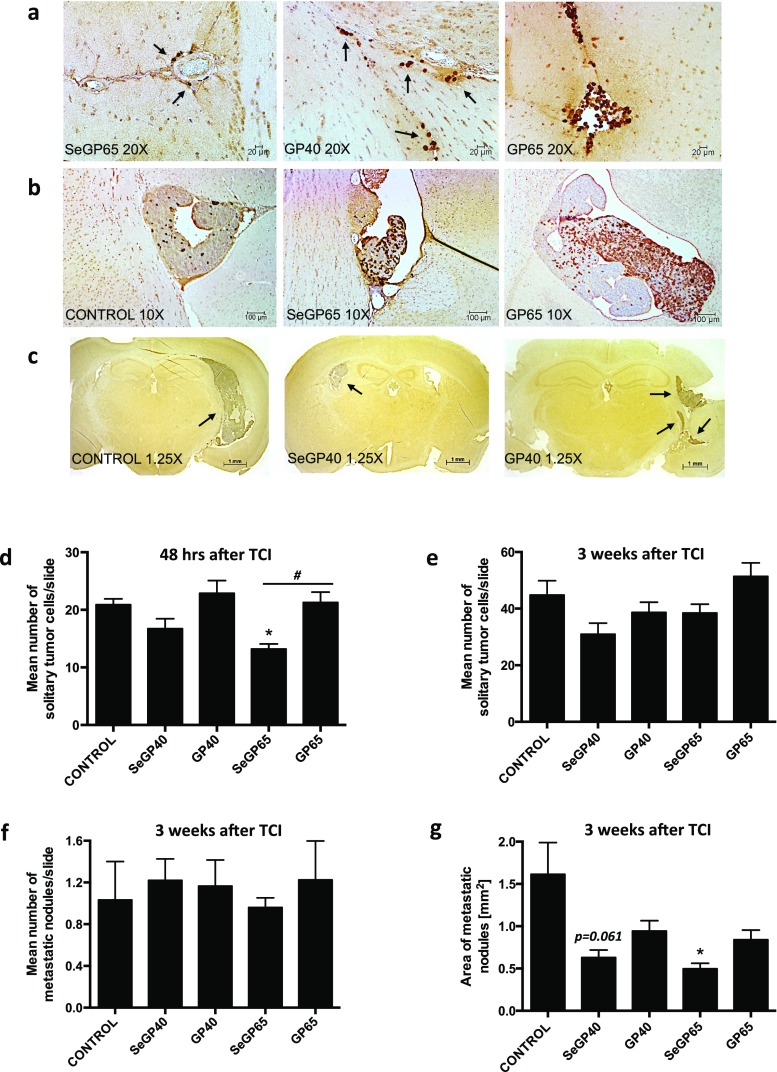
Immunohistochemical analysis of tumor growth in the brain. Tumor progression was evaluated by staining of brain sections for GFP-positive cells 48 h and 3 weeks post tumor cell infusion (TCI). **a** Representative histological images of solitary tumor cells or tumor cells in small clusters at 20× and 40× magnification 48 h post tumor cell infusion. **b** Representative images of metastatic nodules measured at 10× magnification present mainly in lateral and third ventricles 3 weeks post tumor cell infusion. **c** Representative coronal sections at 1.25× magnification showing metastatic nodules 3 weeks post tumor cell infusion. **a**–**c** *Arrows* indicate GFP-positive cells, clusters, and nodules. **d** Mean number of solitary tumor cells (*per slide*) 48 h and **e** 3 weeks post TCI. **f** Mean number of metastatic nodules (*per slide*) and **g** metastatic area 3 weeks post TCI. Values are mean ± SEM, *n* = 4–6 mice per group. *Significantly different as compared with mice fed control diet at *p < 0.05. *#*Values in the group SeGP65 are significantly different as compared with those in the GP65 group at ^*#*^ *p* < 0.05, one-way ANOVA

To further characterize metastatic progression, the brains were sectioned and stained for GFP-positive cells. Figure 3a–c are representative images of morphological changes observed in the brain at different stages of metastatic tumor growth in selected treatment groups. Specify, Fig. 3a provides representative images of tumor cells attached to the brain endothelium and/or in the perivascular space 48 h post tumor cells infusion. Figure 3b and c show at different magnifications representative images of mature metastatic nodules, growing 3 weeks post tumor cells infusion primarily in lateral and third ventricles. Quantified results of these events, which include all treatment groups, are shown in Fig. 3d–g. Solitary tumor cells or tumor cells in micrometastases were counted 48 h (Fig. 3d) and 3 weeks (Fig. 3e) post tumor cell infusion. At 48 h post tumor cell infusion, there was a significant reduction (∼30 %) in the number of solitary tumor cells in mice fed SeGP65 enriched diet as compared with mice fed control diet and infused with tumor cells (Fig. 3d). Furthermore, there was a significant reduction (∼30 %) in the number of solitary tumor cells in SeGP65 fed mice as compared to GP65 fed mice, suggesting a significant contribution of Se. Following 3 weeks of tumor growth, there were no significant differences in the number of solitary tumors cells or the number of metastatic nodules among mice fed any of the experimental diets (Fig. 3e and f). SeGP40 showed trends towards reduced number of solitary tumor cells (Fig. 3e) and reduction of metastatic nodules (Fig. 3g); however, the results did not reach significance. Importantly, feeding mice with SeGP65-enriched diet significantly reduced the size of metastatic nodules by more than 50 % (Fig. 3g).

### Supplementation with Selenoglycoprotein Fractions Alters Gene Expression of Selected Adhesion Molecules in Brain Microvessels

**Fig. 4:**
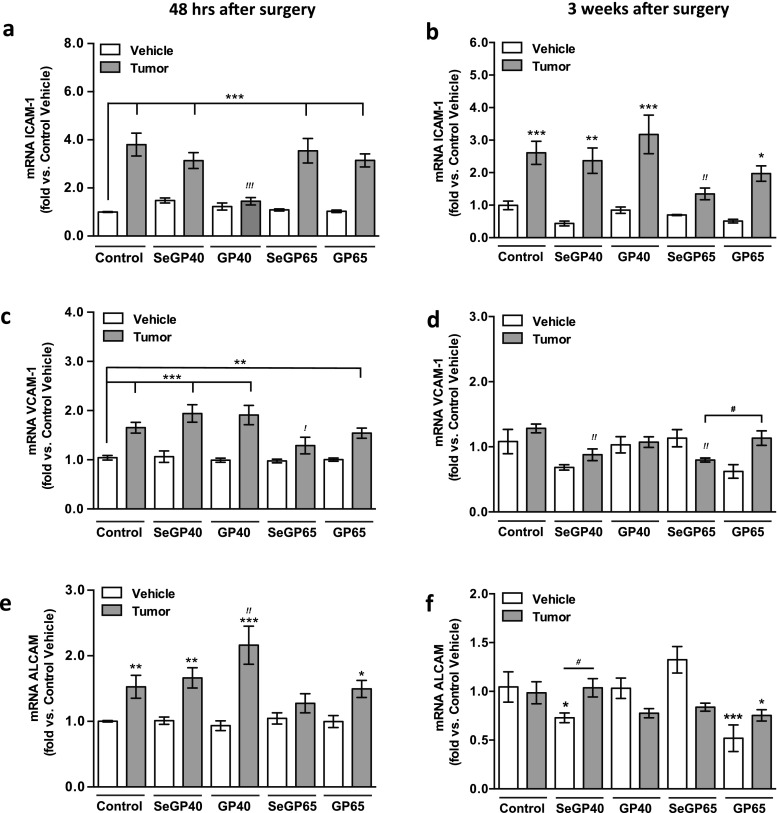
Supplementation with selenoglycoprotein fractions alters gene expression of selected adhesion molecules in brain microvessels. mRNA levels of adhesion molecule following dietary supplementation and tumor cell infusion as measured by RT-PCR. ICAM-1 mRNA levels **a** 48 h and **b** 3 weeks post surgery. VCAM-1 mRNA levels **c** 48 h and **d** 3 weeks post tumor cell or vehicle infusion. ALCAM mRNA levels **e** 48 h and **f** 3 weeks post tumor cell or vehicle infusion. Values are mean ± SEM, n = 4–7 mice per group. Significantly different as compared with control vehicle mice at ***p < 0.001, **p < 0.01 or *p < 0.05. Significantly different as compared with control tumor mice at ^*!!!*^ *p* < 0.001, ^*!!*^ *p* < 0.01 or ^*!*^ *p* < 0.05. Significantly different as indicated at ^#^ *p* < 0.05, two-way ANOVA

The effects of selenoglycoproteins and tumor cell infusion on the expression of selected cell adhesion molecules, namely ICAM-1, VCAM-1, and ALCAM, were evaluated by quantitative reverse transcription PCR. Infusion of tumor cells significantly upregulated ICAM-1 mRNA levels within all dietary treatments except for GP40 (48-h time point) and SeGP65 (3 week time point) (Fig. 4a–b). There was also a significant interaction between diets and tumor cell infusion on ICAM-1 mRNA expression at 48 hr. VCAM-1 mRNA levels were similarly upregulated with tumor cell infusion for all diets except SeGP65 at 48 h post tumor cell infusion (Fig. 4c). Following 3 weeks of tumor growth, VCAM-1 mRNA levels were at the control levels in mice fed all experimental diets, except the SeGP65 fed group, which exhibited a significant decrease in VCAM-1 mRNA levels compared with both control tumor and the GP65 control (Fig. 4d). There was a significant interaction between dietary treatment and tumor cell infusion on VCAM-1 mRNA expression. ALCAM mRNA levels showed significant upregulation at a 48-h time point in the tumor cell infused mice for all dietary group except the SeGP65 fed group, where the differences were not significant (Fig. 4e). ALCAM expression was not changed 3 weeks post tumor cell infusion for all dietary treatments except SeGP40, which increased expression of this adhesion molecule (Fig. 4f). At both 48 h and 3 weeks post tumor cell infusion, there was a significant interaction between diet and tumor cell infusion on ALCAM mRNA levels.

### Selenoglycoprotein Fraction SeGP65 Decreases Expression of Acetylated NF-κB p65 in Brain Microvessels 48 h Post Tumor Cell Infusion

**Fig. 5:**
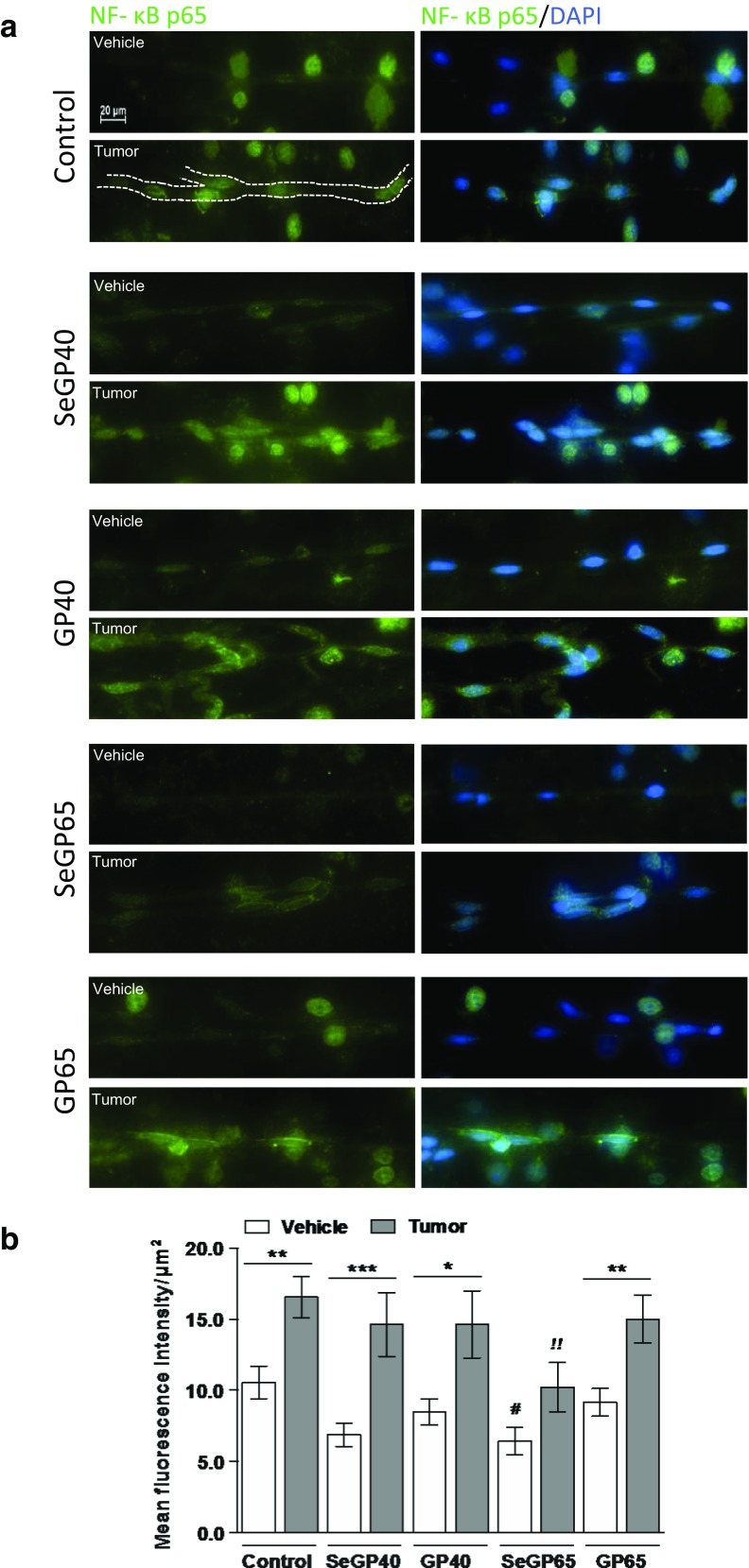
Selenoglycoprotein fraction SeGP65 decreases expression of acetylated NF-κB p65 in brain microvessels 48 h post tumor cell infusion. **a** Representative immunofluorescent images of brain microvessels isolated 48 h after tumor cell or vehicle infusion, stained for NF-κB p65 (acetyl Lys-310) Alexa Fluor488 (green), and DAPI (blue) counterstaining. **b** Quantified fluorescent signal measured from the nuclei of isolated microvessels. Images were acquired using a 40× lens. Values are mean ± SEM, *n* = 3–4 mice per group. Significantly different as compared between vehicle and tumor within dietary treatment at ***p < 0.001, **p < 0.01, or *p < 0.05. Significantly different as compared with control tumor mice at ^*!!*^ *p* < 0.01. Significantly different as compared with control vehicle mice at ^*#*^ *p* < 0.05, two-way ANOVA

The effects of Se-containing compounds and tumor cell infusion on the expression of acetylated NF-κB p65 in brain microvessels were assessed by immunofluorescence microscopy (Fig. 5a–b). The employed method allows to obtain microvessel-enriched fraction, with some minimal contamination from other cell types. Example of a microvessel was outlined in Fig. 5a. Translocation of acetylated NF-κB p65 only to the nuclei associated with microvessels was quantified. The nuclei were stained by DAPI and the right column reflects merged images of acetylated NF-κB p65 and DAPI.

Infusion of tumor cells significantly increased (by ∼50–100 %) expression of NF-κB p65 (acetyl Lys-310) within all dietary treatments, except for SeGP65. Instead, mice fed SeGP65 enriched diet and infused with tumor cells displayed significant reduction (by ∼60 %) in expression of acetylated form of NF-κB p65 in brain microvessels as compared to control tumor group (Fig. 5b). Furthermore, SeGP65 vehicle group showed significant decrease (by ∼40 %) in expression of NF-κB p65 (acetyl Lys-310) when compared with vehicle-treated control (Fig. 5b).

## Discussion

Diet and physical activity are crucial behavioral factors modulating cancer risk and possibly affecting tumor progression [26, 27]. Previously, we investigated a nutritional-based intervention specifically targeting adhesion and tumor cell migration of tumor cells in vitro. We demonstrated effectiveness of specific selenoglycoproteins to diminish these events and inhibit early stages of cancer progression [22]. Based on these findings, we explored in the present study whether selenoglycoproteins incorporated into standard chow could modulate brain metastatic progression in mice.

Initially considered a toxic and potentially carcinogenic element, Se was recognized in the second half of the twentieth century as an essential nutrient with a significant role in human health and well being [28, 29]. It has been reported by numerous epidemiological studies that low Se status is associated with increased risk of certain types of cancer in terms of incidence and mortality [30–32]. Indeed, anticancer properties of Se have been demonstrated in various laboratory studies, including carcinogenesis experiments based on different animal models, with most of them showing a significant decrease in cancer incidence [33, 34]. In spite of the scientific evidence demonstrating the anticancer properties of Se, inconsistent results from clinical trials have lessened the initial recommendations for Se supplementation [19, 35, 36]. Recently, it has been pointed out that the contradictory findings might be due to the lack of understanding of the mechanisms underlying Se biology [37]. Furthermore, growing evidence indicates that anticancer effects of Se supplementation depend not only on the dose and biological form of Se and cancer type, but also on the baseline Se status of the population. Beneficial effects of Se supplementation should be expected only when the Se levels prior to supplementation are low or suboptimal [13, 38, 39].

While most of the studies have investigated the relation of Se and cancer prevention, distinctly less attention has been given to cancer progression and metastasis. The metastatic model employed in our study was based on the infusion of Lewis lung carcinoma cells into the brain microvasculature, as lung cancers represent one of the most common primary tumors creating secondary lesions in the brain [3, 40]. The results indicate that supplementation with specific selenoglycoproteins can modulate tumor cell homing in mouse brain. Tumor progression in the brain as measured by in vivo imaging as well as post hoc immunohistochemical staining revealed similar trends implicating Se as a potential regulator of the metastatic process. Indeed, previous reports have indicated attenuation of lung and liver metastatic tumors growth following Se supplementation [41–43].

It has been shown that the beneficial effects of Se on cancer metastasis largely depend on the chemical form [11, 41]. Although Se can be supplemented as organic or inorganic compounds, each possessing anticancer properties, the inorganic forms of Se show lower rates of absorption, tissue accumulation, and antioxidant bioavailability as compared to organic forms [44]. In our previously published study [18], we compared the properties of several Se compounds, including L-selenomethionine, sodium selenite, and Se-enriched yeast formulation, on the development of melanoma brain metastatic lesions. Our results indicated that mice bearing brain metastatic tumors and fed Se-yeast-enriched diet displayed a higher survival rate and decreased tumor growth as compared to controls. Another study revealed that selenomethionine could attenuate or delay breast cancer metastasis in mice, whereas selenite did not provide protection [41]. These protective effects of organic Se-yeast formulation, where selenomethionine was the primary source of Se, as well as selenomethionine itself, may be attributed to non-specific incorporation of selenomethionine into the general body proteins. Since selenomethionine is readily stored in the organism, treatment with selenomethionine provides an advantage over dietary supplementation with other Se compounds [44, 45]. For example, it has been demonstrated that selenomethionine-supplemented animals maintained higher activity of selenoenzymes during Se depletion for longer periods than selenite-supplemented animals [45]. In our study, both SeGP40 and SeGP65 were extracted from Se-enriched yeast and contain mostly organic Se [18, 22]. The process of SeGP extraction was designed to capture specific water-soluble SeGP fractions and was based on the main pH ranges of these species [22]. This distinct composition of SeGP40 and SeGP65 translates into their different biological activities. Although SeGP40 showed some trends towards reduced brain metastatic tumor growth, the results did not reach significance. It is likely that distinct SeGPs employ different molecular pathways that lead to modulation of tumor progression in the brain.

Consistent with reports suggesting that the form of Se plays a role, the present study demonstrates that supplementation with selected selenoglycoproteins, mainly SeGP65, can modulate mRNA levels of specific pro-inflammatory adhesion molecules. The adhesion molecules evaluated in this study, namely ICAM-1, VCAM-1 and ALCAM, are known to be involved in tumor cell attachment to the vascular endothelium and are directly implicated in the progression of tumor growth, including early stages of metastasis seeding in the brain [3, 46]. In the early stage of tumor progression, i.e., 48 h post tumor cell infusion; all studied adhesion molecules were significantly upregulated (as much as fourfold). Only microvessels isolated from SeGP65 supplemented mice showed no difference with tumor cell infusion in VCAM-1 and ALCAM mRNA levels, indicating a potential protective effect. Importantly, the positive effects of SeGP65 were also maintained in mice with mature tumors, i.e., 3 weeks of tumor growth, when ICAM-1 mRNA levels were still elevated within most treatment groups and SeGP65 fed mice alone showed no change with tumor cell infusion.

Interesting results were observed when analyzing the impact of GP40 supplementation on tumor cell-induced upregulation of ICAM-1. GP40 was the only fraction which protected against ICAM-1 mRNA increase 48 h post tumor cell infusion. However, these effects were not sustained following 3 weeks of tumor growth, with ICAM-1 mRNA levels being highly elevated in GP40-fed mice with mature tumors. Based on these observations, the impact of specific GPs and SeGPs on expression of ICAM-1 in brain microvessels appears to be dependent on the stage of tumor growth. Furthermore, these results suggest a potential role of the glycoprotein component in modulating the adhesive properties of brain microvasculature. While the most effective in modulating tumor cell progression in the brain were Se containing fractions, the current results as well as our previously published data [22] suggest the role of not only Se, but also glycoproteins in the observed effects.

An inducible transcription factor, NF-κB is a key regulator of inflammatory, immune, and antiapoptotic responses in mammalian cells [47]. To reach full biological activity NF-κB must undergo several post-translational modifications, including intranuclear, reversible acetylation of distinct lysine residues in the p65 subunit [48]. Acetylation of p65 is crucial for modulating NF-κB nuclear functions and has been shown to enhance its DNA binding and transcriptional activities. In contrast, deacetylation stimulates binding of endogenous inhibitor IκBα and promotes rapid nuclear export of the NF-κB complex [48–50]. In our current study infusion of tumor cells robustly increased the acetylation status of NF-κB p65 in the nuclei of isolated microvessels. Importantly, supplementation with SeGP65, but not glycoprotein fractions, significantly reduced baseline acetyl-NF-κB p65 expression and attenuated tumor cell-induced activation of this transcription factor. These results are consistent with the impact of SeGP65 on the expression of adhesion molecules evaluated in this study. Indeed, NF-κB is known to play an essential role in pro-inflammatory activation of human endothelium, including expression of cell adhesion molecules [51, 52].

Tumor cells are known to produce high levels of reactive oxygen species (ROS) [53]. We also reported that extravasation and growth of brain metastatic tumors are related to oxidative stress [26]. It has been demonstrated that pro-inflammatory activation of vascular endothelium, characterized by activation of various transcription factors, including NF-κB, and upregulation of adhesion molecules, results from an increase in ROS [54]. Selenium is a crucial cofactor for several important antioxidative enzyme systems, including glutathione peroxidase (GPx), and anticancer effects of Se are largely attributed to its ability to modulate oxidative stress [12, 14, 53]. GPx, along with several other selenoproteins, have been detected in human and rodent brains [9, 55] and may modulate, at least in part, the effects observed in the present study. To support this notion, it has been reported that GPx deficiency stimulates expression of various adhesion molecules on the endothelial cell surface [54, 56].

In conclusion, our study indicates that specific selenoglycoproteins may be considered as possible candidates for adjuvant therapies aiming at suppression of brain metastatic tumor growth by modulating adhesive properties of brain vascular endothelium. These results further warrant the development of novel tumor targeted Se compounds or supplements tailored for cancer and metastasis prevention.
